# Sugemalimab combined with chemotherapy for the treatment of advanced esophageal squamous cell carcinoma: a cost-effectiveness analysis

**DOI:** 10.3389/fphar.2024.1396761

**Published:** 2024-06-28

**Authors:** Hongfu Cai, Ling Fang, Zhiwei Zheng

**Affiliations:** ^1^ Department of Pharmacy, Fujian Medical University Union Hospital, Fuzhou, China; ^2^ Department of Pharmacy, Cancer Hospital of Shantou University Medical College, Shantou, China

**Keywords:** esophageal squamous cell carcinoma, sugemalimab, chemotherapy, cost-effectiveness, partitioned survival approach model

## Abstract

**Background:**

This study aims to systematically analyze the cost-effectiveness of the combination therapy comprising sugemalimab and chemotherapy in the management of advanced ESCC from the Chinese healthcare system perspective.

**Methods:**

An advanced ESCC patient simulation partitioned survival approach model was developed to mimic the disease progression of patients undergoing treatment with sugemalimab in combination with chemotherapy *versus* chemotherapy alone. To ensure accuracy and precision, clinical data, treatment costs, and utility values were collected from comprehensive clinical trials and reliable economic databases. The cost-effectiveness analysis was conducted by assessing the incremental cost-effectiveness ratio in relation to the established willingness-to-pay threshold. One-way and probabilistic sensitivity analyses were performed to assess the robustness of the model.

**Results:**

The cumulative expenditure for the group of patients administered with sugemalimab amounted to US$ 41734.87, whereas the placebo group was associated with a total cost of US$ 22926.25. By evaluating the ICER, which quantifies the additional cost incurred per QALY gained, a value of US$ 61066.96 per QALY was determined. It is imperative to note that this ICER value surpasses the predetermined threshold for WTP in China, set at US$ 39,855.79 per QALY. Sensitivity analyses demonstrated that the results were sensitive to the cost of sugemalimab, progression-free survival, and utility values. These fluctuations did not result in a reversal of the study findings.

**Conclusion:**

The combination of sugemalimab with chemotherapy for the treatment of ESCC in China is currently not considered a cost-effective therapeutic approach. However, it is suggested that additional reductions in price may facilitate the potential for achieving cost-effectiveness.

## 1 Introduction

Esophageal cancer is recognized as one of the most prevalent forms of cancer worldwide, ranking seventh in terms of incidence and sixth in terms of mortality (Jiang et al., 2023). Disturbingly, China bears the greatest burden of this disease, accounting for approximately 50% of all global esophageal cancer cases and deaths each year (Li L. et al., 2022). Esophageal squamous cell carcinoma (ESCC) is an extremely aggressive and deadly malignancy, predominantly found in Eastern Asia, primarily in China (Morgan et al., 2022). It poses a significant health burden in the region, warranting immediate attention. The prognosis for advanced ESCC is exceedingly grim, with a 5-year survival rate of less than 20% (Zhu et al., 2023). Consequently, urgent action is required to devise effective and precise therapeutic approaches to combat this devastating disease. The treatment of advanced ESCC is challenging due to late-stage diagnosis, tumor invasion into adjacent tissues, and distant metastasis. Current therapeutic approaches, including surgery, chemotherapy, and radiation therapy, have limited efficacy, and the development of resistance to these treatments is a common occurrence (Yajima et al., 2021). Therefore, there is a critical need to explore new treatment options that can improve the survival and quality of life for patients with advanced ESCC.

The emergence of immunotherapy has revolutionized the field of cancer treatment (Thuss-Patience and Stein, 2022; Wu et al., 2023). In particular, immune checkpoint inhibitors (ICIs), such as programmed cell death protein 1 (PD-1) and its ligand PD-L1, have demonstrated significant promise in the treatment of various cancers, including ESCC. Several recent clinical trials have provided compelling evidence of the efficacy of immune checkpoint inhibitors in the treatment of advanced ESCC. One notable ICIs that has demonstrated promising results in the treatment of advanced ESCC is pembrolizumab, as shown in the KEYNOTE-590 trial (Sun et al., 2021). This randomized, phase 3 trial investigated the efficacy of pembrolizumab in combination with chemotherapy compared to chemotherapy alone as the first-line treatment for patients with advanced ESCC. The results revealed a significant improvement in overall survival, progression-free survival, and objective response rate in the pembrolizumab group, highlighting its potential as a valuable treatment option for these patients. Similarly, nivolumab, another immune checkpoint inhibitor targeting PD-1, has demonstrated encouraging outcomes in advanced ESCC. The CheckMate 648 trial investigated the efficacy of nivolumab plus chemotherapy as first-line treatment for patients with unresectable advanced or metastatic ESCC (Doki et al., 2022). The trial revealed a significant improvement in overall survival and response rates compared to chemotherapy alone, further supporting the use of immune checkpoint inhibitors in the management of advanced ESCC. Recently, the use of sugemalimab, a novel anti-PD-L1 antibody, has shown great promise as a therapeutic option for patients with advanced ESCC. The GEMSTONE-304 trial evaluated the efficacy and safety of sugemalimab as first-line treatment in patients with advanced ESCC(Li et al., 2024). The results of the GEMSTONE-304 trial demonstrated significant improvements in overall survival (OS), progression-free survival (PFS), and response rates in patients receiving sugemalimab compared to those receiving chemotherapy alone. The median OS was substantially prolonged in the sugemalimab group, indicating a consistent and remarkable survival benefit. Moreover, the sugemalimab combination therapy exhibited a higher objective response rate and disease control rate compared to chemotherapy alone, further highlighting its efficacy in combating ESCC.

However, despite the promising initial results, there is a dearth of comprehensive evaluation regarding the cost-effectiveness of sugemalimab in combination with chemotherapy when compared to chemotherapy alone. Cost-effectiveness analyses are essential tools in healthcare decision-making processes, as they provide a comprehensive evaluation of treatments by considering not only their clinical effectiveness but also their cost implications (Rodriguez and Caughey, 2013). Healthcare decision-making is often confronted with limited resources, and cost-effectiveness analyses assist in prioritizing interventions based on the potential returns on investment. By comparing the costs and outcomes of different treatment options, decision-makers can identify interventions that provide the greatest health gains at a reasonable cost (Turner et al., 2021). This helps promote efficient resource allocation and ensures equitable access to healthcare services. Thus, the lack of cost-effectiveness has hindered our understanding of the economic implications of utilizing sugemalimab therapy for advanced ESCC. In this comprehensive study, our objective is to evaluate the incremental cost-effectiveness ratio (ICER) of combining sugemalimab with chemotherapy, as compared to the use of chemotherapy alone. This analysis will offer critical insights into the financial implications associated with achieving an additional unit of health benefit through the adoption of sugemalimab in combination with chemotherapy. The outcomes of this study will serve as a valuable resource for decision-makers, who are tasked with allocating limited resources efficiently. The results will aid in determining the optimal allocation of healthcare funds while considering both the financial sustainability and clinical effectiveness of treatment options.

## 2 Methods

### 2.1 Study design and data sources

**FIGURE 1:**
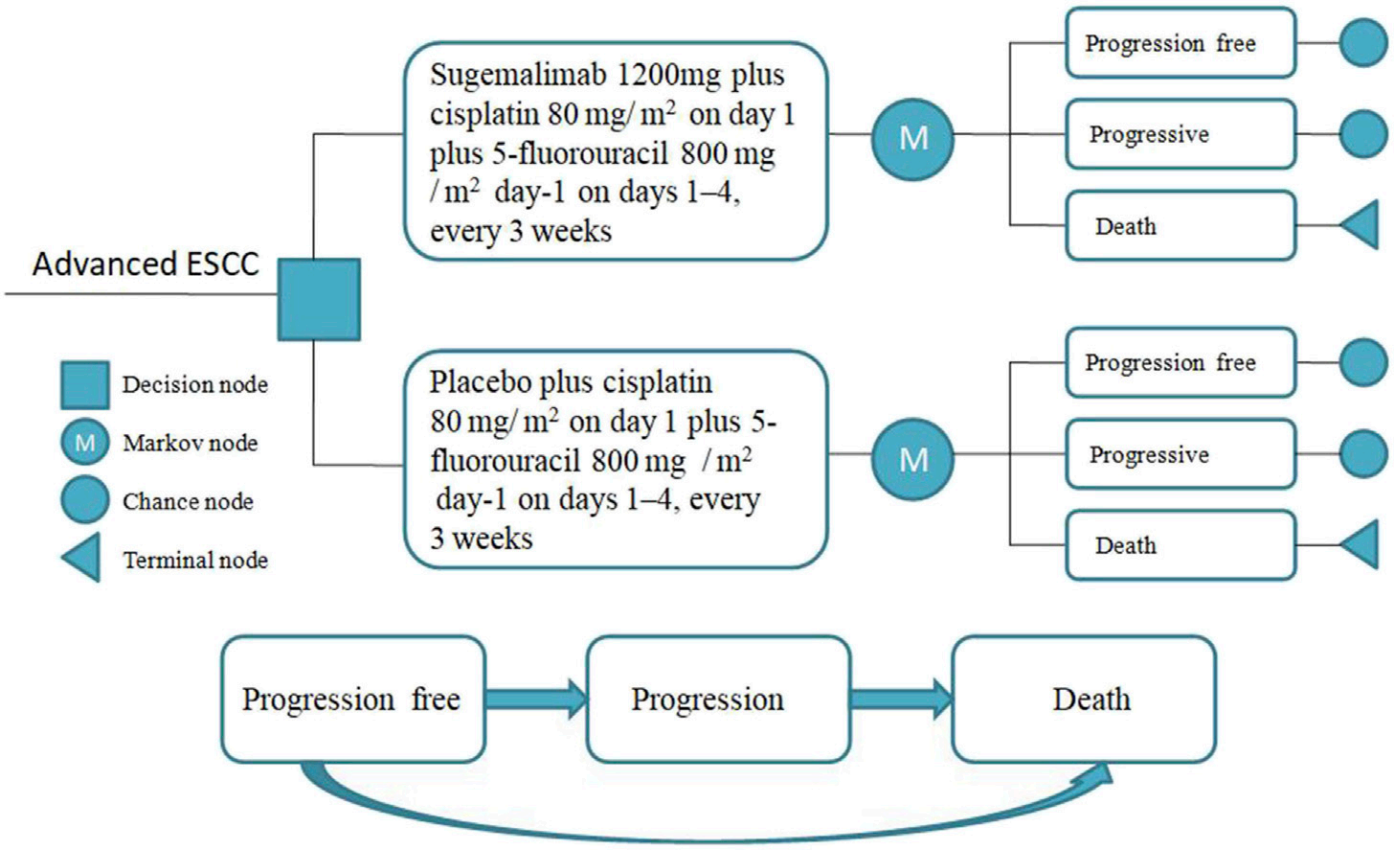
Study design model.

We have developed a novel partitioned survival model (PSM) to comprehensively assess the intricate relationship between cost aspects and clinical benefits in patients with advanced ESCC. The PSM categorizes patients into three distinct and mutually exclusive states: progression-free disease, progressive disease, and death (Figure 1). By incorporating a wide range of significant direct healthcare expenditures, including medication costs, management of adverse events, follow-up therapeutic interventions, and optimal supportive care, our model provides a comprehensive framework for evaluating the economic implications of ESCC treatment.

Survival data from the GEMSTONE-304 clinical trial was collected using GetData Graph Digitizer (version 2.25) software. This software reconstructs the raw data from survival curve prior curves to simulate transfer probabilities.

To align with the designated observation time of the GEMSTONE-304 clinical trial, we have selected a simulation period of 21 days per cycle. Because the overall 5-year survival rate for ESCC ranges was 20%, we set the time horizon of our model to 10 years. Furthermore, our PSM analysis provides additional support for this 10-year timeframe by demonstrating that nearly all patients in the model would reach the terminal state within this duration.

According to the latest China Guidelines for Pharmacoeconomic Evaluations in 2020, we have established a threshold for willingness-to-pay (WTP) at $39855.785 per quality-adjusted life year (QALY), which was determined as three times the national gross domestic product (GDP) *per capita* in 2023 (National Bureau of Statistics of China, 2023; Liu et al., 2020). The incorporation of this WTP threshold enables us to compare the incremental cost per QALY gained and assess the economic viability of the intervention. The construction of the PSM framework was executed using TreeAge Pro 2011 software.

## 3 Population and intervention

The model assumes that the study population is consistent with participants in the GEMSTONE-304 trial. Between 19 December 2019, and 23 December 2021, a total of 785 patients were screened for eligibility across 69 study centers in China. Out of these patients, 540 individuals who met the criteria were randomly assigned to two groups in a 2:1 ratio. Specifically, 358 patients were allocated to receive sugemalimab in conjunction with chemotherapy, while 182 patients were assigned to receive a placebo alongside chemotherapy.All enrolled patients were of Asian ethnicity and had a median age of 62.5 years (range, 40–75 years) and 63.0 years (range, 43–75 years) in the sugemalimab and placebo-chemotherapy groups, respectively. The majority of patients had an Eastern Cooperative Oncology Group (ECOG) performance status score of 1 (79.1% in the sugemalimab group *versus* 78.6% in the placebo-chemotherapy group). The proportion of male patients was similar between the two groups, with 87.7% in the sugemalimab group and 86.8% in the placebo-chemotherapy group. The majority of patients in both treatment groups had stage IV tumors (90.2% in the sugemalimab group *versus* 90.7% in the placebo-chemotherapy group), and a similar proportion of patients had metastasis (79.6% in the sugemalimab group *versus* 79.1% in the placebo-chemotherapy group). The distribution of patients based on PD-L1 expression levels was balanced in both treatment groups, with 57.0% and 57.1% of patients having PD-L1 expression levels below 10% and 43.0% and 42.9% having PD-L1 expression levels of 10 or higher in the sugemalimab and placebo-chemotherapy groups, respectively (Li et al., 2024).

The GEMSTONE-304 trial and our PSM assumes that the study population included a total of 540 eligible patients who were randomly assigned in a 2:1 ratio to receive either sugemalimab along with chemotherapy (*n* = 358) or placebo along with chemotherapy (*n* = 182). Participants were randomly allocated to receive either sugemalimab (1,200 mg) every 3 weeks or a placebo for a maximum of 24 months. Additionally, they will receive chemotherapy every 3 weeks, comprising cisplatin (80 mg/m^2^, day 1) and 5-fluorouracil (800 mg/m^2^, day 1–4), for a total of six treatment cycles.

The administration of subsequent anticancer therapy was observed in 145 individuals (40.5%) within the sugemalimab-chemotherapy cohort, whereas 88 participants (48.4%) received comparable treatment in the placebo chemotherapy group. In light of the cost-effectiveness analysis that needs to be conducted, we have employed the assumption of utilizing a second-line treatment of docetaxel in combination with a platinum-based chemotherapy regimen for both treatment groups. However, due to the considerable uncertainty surrounding the optimal selection of third-line therapy, our study assumes the utilization of the best supportive treatment regimen in the event of disease re-progression.

## 4 Clinical data collection procedures

In this study, we extracted and reconstructed the survival curve data from the GEMSTONE-304 clinical trial using the data extraction tool GetData Graph Digitizer (http://www.getdata-graph-digitizer.com, version 2.25) software.Our primary aim was to extract and simulate the survival curves, encompassing both overall survival and disease-free survival, by identifying the most suitable statistical distributions. In the determination of the optimal distribution, a combination of two principles was employed: firstly, the akaike information criterion (AIC) and the Bayesian Information Criterion (BIC) were utilized as the minimum statistical criteria, and secondly, an intuitive visual inspection was conducted to ensure that the simulated curves adhered to the established clinical observation standards (Hoyle and Henley, 2011). The AIC and BIC values associated with the simulated survival curves can be located in Supplementary Table S1. Furthermore, Supplementary Figure S1 provides a graphical depiction of the reconstructed distribution curves for each respective group.

To enhance the efficacy of our model, we implemented a simulation approach to generate survival times based on a log-logistic distribution. This innovative technique allowed us to extend the applicability of our model beyond the duration of the clinical trial follow-up. By providing a robust estimation of the survival function S(t), we aimed to offer a more comprehensive understanding of the underlying dynamics of the studied population. The survival function, denoted as S(t), plays a crucial role in survival analysis as it quantifies the probability that an individual survives beyond a given time point, t. In our study, we employed the log-logistic distribution to model the survival function,S(t) = 1/(1+λt^γ^). This parametric distribution holds immense significance in survival analysis due to its flexibility in capturing various shapes of survival curves.To effectively implement the log-logistic distribution and estimate the parameters, we utilized R software. The estimated values of the parameters, shape (γ) and scale (λ) are presented in Table 1.

**TABLE 1:** Log-logistic survival model parameters.

Variable	Sugemalimab plus chemotherapy cohort	Placebo plus chemotherapy cohort
Log-logistic OS shape (γ)	1.707	1.975
Log-logistic OS scale (λ)	0.00937	0.00773
Log-logistic PFS shape (γ)	2.129	2.501
Log-logistic PFS scale (λ)	0.0173	0.0166

Abbreviations:OS: overall survival; PFS: Progression-Free Survival.

## 5 Cost sources and utility parameters

This study is conducted from the Chinese healthcare system perspective. From this perspective, it can provide decision-making for healthcare and promote rational pricing of medicines. We consider costs related to medication, treatment of serious adverse events caused by medications, follow-up treatment, best supportive care, and expenses related to follow-up. The annual exchange rate from Chinese Renminbi (RMB) to US dollars (US$) for 2023 was705 units of RMB per 100 US$(National Data, 2023).To ensure accurate data on drug costs, we gathered national median drug prices from the China Data Platform (https://data.yaozh.com/) (YaoZH, 2024). Conversely, other costs were derived from pertinent literature that has been previously published.

In order to evaluate the quality of life associated with health status, utility values ranging from 0 to 1 were utilized within this study. Unfortunately, explicit utility value data from the GEMSTONE-304 clinical trial were unattainable. Consequently, we procured utility values from previously published literature. It is important to highlight and emphasize that these cost and utility values obtained from the literature are factored into our sensitivity analysis, which aims to determine the robustness of the results yielded by our model by assessing their impact on the conclusions drawn from our findings. Additionally, our model also accounts for the negative utility associated with adverse drug events. Detailed information regarding cost and utility values can be found in Table 2.

**TABLE 2:** The parameters input of the model.

Parameters	Input value	Range		Distribution	Source
		Minimum	Maximum		
TEAE rate of sugemalimab group (%)					
Platelet count decreased	2.50	-	-	Beta	Li et al. (2024)
Vomiting	2.00			Beta	Li et al. (2024)
Nausea	0.80	-	-	Beta	Li et al. (2024)
TEAE rate of placebo group (%)					
Platelet count decreased	2.20	-	-	Beta	Li et al. (2024)
Vomiting	2.10	-	-	Beta	Li et al. (2024)
Nausea	2.20	-	-	Beta	Li et al. (2024)
Medication costs (US$)					
Sugemalimab (600 mg)	1839.85	1379.89	2299.81	Gamma	YaoZH (2024)
Fluorouracil (500 mg)	29.12	21.84	36.40	Gamma	YaoZH (2024)
Cisplatin (10 mg)	1.39	1.04	1.74	Gamma	YaoZH (2024)
Cost of TEAE per cycle (US$)					
Platelet count decreased	1523.82	1142.87	1904.78	Gamma	Liu et al. (2023a)
Vomiting	71.00	53.25	88.75	Gamma	Li et al. (2022b)
Nausea	101.15	75.86	126.44	Gamma	Yang et al. (2021)
Subsequent therapy per cycle (US$)	639.75	479.81	799.69	Gamma	Zheng et al. (2024)
Best supportive care (US$)	182.23	136.67	227.79	Gamma	Liu et al. (2023b)
Follow-up cost per cycle (US$)	73.72	55.29	92.15	Gamma	Liu et al. (2023a)
Utility					
Progression-free disease	0.74	0.56	0.93	Beta	Al-Batran et al. (2016)
Progressive disease	0.58	0.44	0.73	Beta	Al-Batran et al. (2016)
Platelet count decreased	0.20	0.15	0.25	Beta	Zheng et al. (2023a)
Vomiting	0.13	0.10	0.16	Beta	Zheng et al. (2023a)
Nausea	0.13	0.10	0.16	Beta	Zheng et al. (2023b)
Body surface area (m^2^)	1.72	1.29	2.15	Beta	Zheng et al. (2023b)
Discount rate	5%	0	8%	Beta	Liu et al. (2020)

Abbreviations: TEAE:Treatment-emergent adverse event.

## 6 Sensitivity analysis

This study utilized sensitivity analyses to enhance the robustness of the model. Firstly, a one-way sensitivity analysis was conducted to assess the impact of varying input parameters on the ICER. Each input parameter was adjusted individually by ±25% to evaluate its influence on the ICER. Additionally, the discount rate was varied from 0% to 8%. The results of this sensitivity analysis were visually represented using a tornado diagram, an effective tool for illustrating the relative importance of each parameter in influencing the ICER.

In order to thoroughly evaluate and quantify the uncertainty associated with estimating the ICER, a comprehensive and rigorous probabilistic sensitivity analysis was carried out. This analysis entailed the execution of 1000 Monte Carlo simulations, allowing for a thorough exploration and investigation of a wide spectrum of probabilistic scenarios. The main objective of the probabilistic sensitivity analysis was to incorporate random sampling of input parameters from specified probability distributions. This approach ensured a robust and comprehensive assessment of the potential variability in the ICER estimates, taking into account the intrinsic uncertainty associated with each parameter. By sampling from specified probability distributions, the analysis allowed for the consideration of parameter values that were not only deterministic, but also probabilistic in nature. This feature enabled a more realistic and accurate representation of the inherent uncertainty in the estimation of the ICER, considering the various sources of variability and randomness that influence the input parameters. The results of this probabilistic sensitivity analysis are effectively communicated through meticulously crafted scatter plots.

## 7 Results

### 7.1 Cost-effectiveness outcomes

The total cost for the group receiving sugemalimab amounted to US$41734.87, whereas the placebo group incurred a total cost of US$22926.25. The administration of the sugemalimab regimen resulted in a statistically significant increase of 0.31 QALYs compared to the placebo group. However, this additional benefit came at an incremental cost of US$18808.62. Consequently, the calculated ICER was US$61066.96 per QALY gained, which exceeds the WTP threshold of US$39855.79 per QALY in China. Thus, the use of the sugemalimab regimen may not be deemed cost-effective within the Chinese healthcare system. Table 3 provides a comprehensive summary of the findings obtained in this analysis.

**TABLE 3:** The results of cost-effectiveness.

Group	Sugemalimab group	Placebo group
Result		
Cost (US$)	41734.87	22926.25
QALYs	1.54	1.23
Incremental cost (US$)	18808.62	NA
Incremental QALY	0.31	NA
ICER (US$/QALY)	61066.96	NA

ICER, Incremental cost–effectiveness ratio; QALY, Quality-adjusted life year; NA, not applicable.

However, we discovered that reducing the price of sugemalimab by 50% to only $919.92 per 600 mg resulted in an ICER of US$39,547.54 per QALY gained. It is noteworthy that this ICER value closely aligns with the WTP threshold of US$39,855.79 per QALY.

## 8 Sensitivity analysis outcomes

**FIGURE 2:**
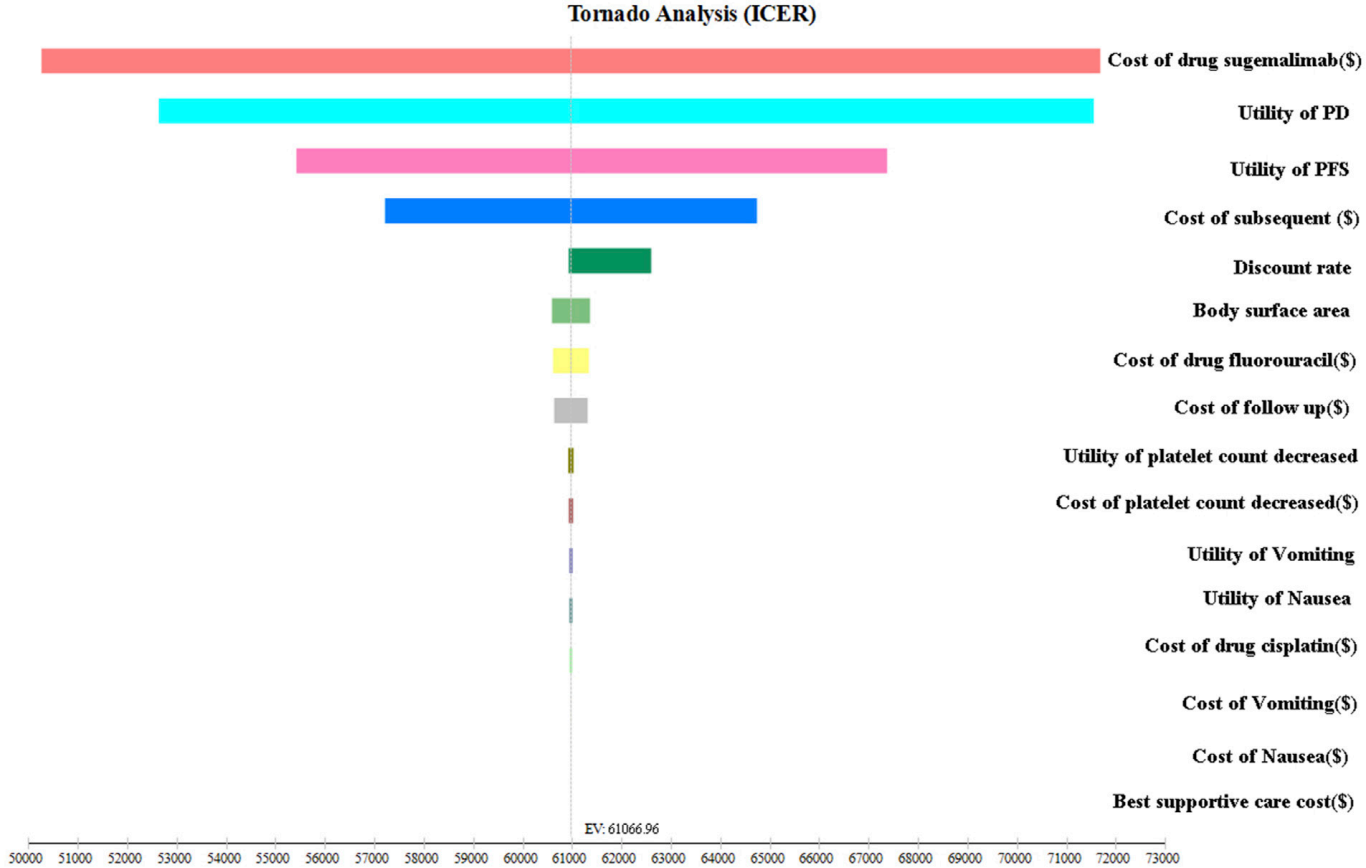
The outcomes of sensitivity analysis.

The tornado diagram presented in Figure 2 depicts the outcomes of one-way sensitivity analysis. The parameter that exerted the greatest impact on the ICER across all populations was the price of sugemalimab. However, it is worth noting that this impact fluctuated within a range of ±25%, which still considerably exceeded the WTP threshold. These fluctuations did not result in a reversal of the study findings. Additionally, other parameters such as PFS utility, PD utility, and Subsequent therapy costs also influenced the ICER, albeit their impact gradually diminished. It is of significance to emphasize that even variations of these parameters within a range of ±25% did not yield significant alterations in the analysis results. Consequently, the consistent finding that the ICER value consistently surpasses three times the GDP strengthens the stability of our findings.

**FIGURE 3:**
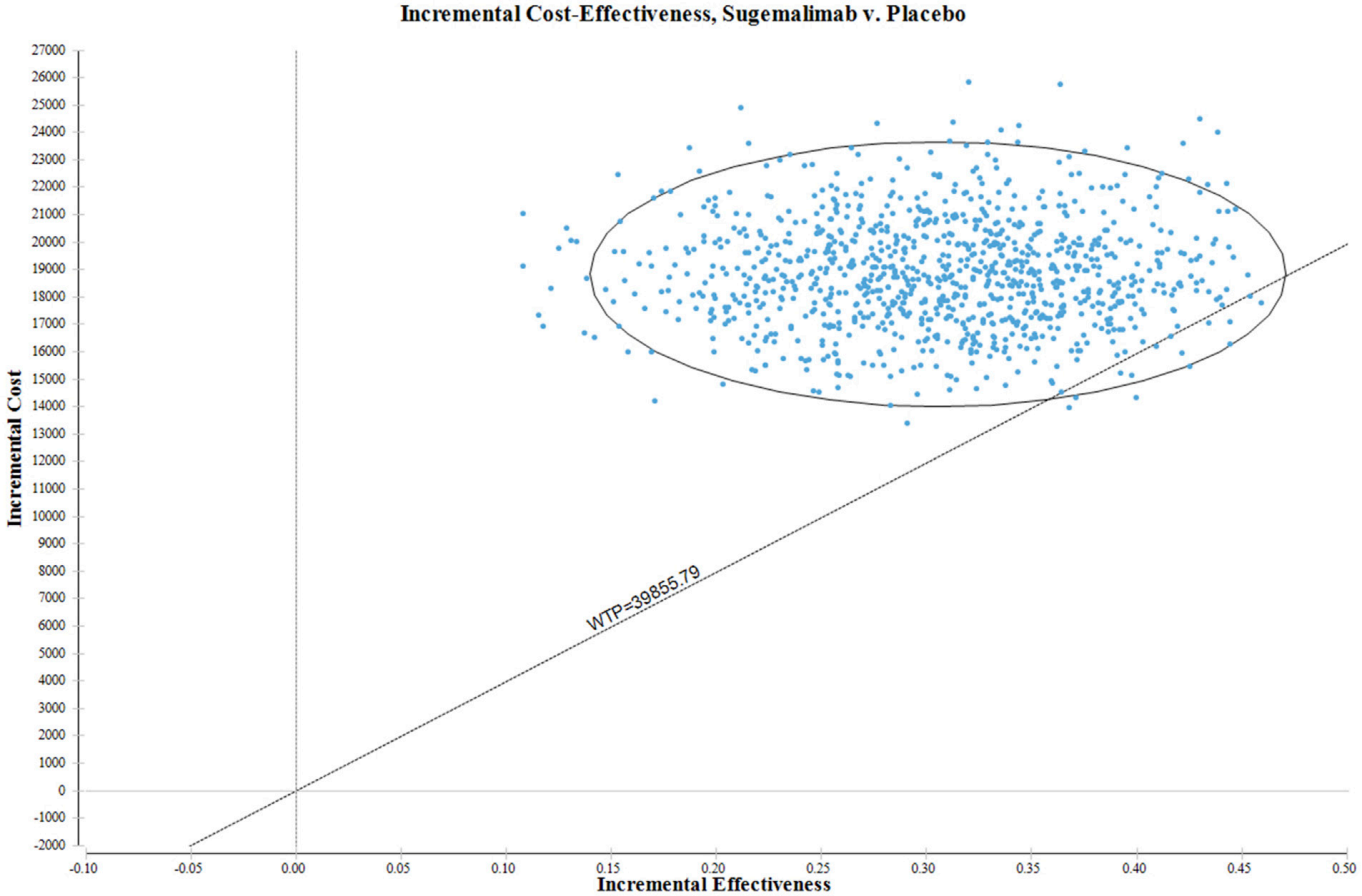
The outcomes of the ICER plane scatter plots.

In Figure 3, the ICER plane is visually partitioned into quadrants, enabling a clear depiction of the distribution of the 1000 bootstrap replicates of the ICER. The resulting graph provides valuable insights into the cost-effectiveness of various interventions. Based on the findings derived from the analysis, interventions falling within the North-East quadrant of the cost-effectiveness plane, specifically below the linear ICER line, are inferred to be perceived as cost-effective. The strategic placement of the North-East quadrant on the cost-effectiveness plane signifies interventions that demonstrate superior cost-effectiveness in comparison to other alternatives. This positioning suggests that the interventions in this quadrant possess a more favorable ICER ratio, indicating lower costs or superior effectiveness relative to interventions in other quadrants. Significantly, when considering the WTP threshold of US$39855.79 per QALY, there is a mere 1.2% probability of deeming the sugemalimab regimen as a more cost-effective option compared to the placebo group.

## 9 Discussion

In recent years, there have been significant advancements in the field of cancer treatment in China, particularly with the development of innovative PD-1 inhibitors. These inhibitors have shown promising results in terms of providing improved survival benefits and clinical tolerability for patients suffering from various types of cancer (Zhang et al., 2022a). One of the key factors contributing to this positive trend is the implementation of centralized price negotiation mechanisms, which aim to enhance the accessibility and affordability of these treatments for patients (Zhang et al., 2022b). The National Medical Products Administration in China has played a crucial role in strengthening regulatory capacity to ensure the safety and efficacy of these innovative anti-cancer medicines. They have also introduced priority procedures to expedite the development, review, and approval of such drugs. These measures have not only accelerated the availability of these treatments but have also facilitated timely access for patients in need.

To address the concern of rapidly increasing healthcare costs, value-based pricing and national medical insurance negotiations have been pivotal in determining the coverage and reimbursement of innovative drugs by the national medical insurance program (Si et al., 2020). This approach ensures that the cost of these medications aligns with the value they provide in terms of patient outcomes and overall healthcare system sustainability. Through these negotiations, drug prices have been reduced by half, thereby significantly easing the financial burden on patients while ensuring the long-term viability of the medical insurance program (Tang et al., 2020). The Chinese government’s concerted efforts in fostering innovation, streamlining regulatory processes, and implementing cost-containment measures have paved the way for the development and accessibility of groundbreaking cancer therapies. These advancements not only offer new hope for cancer patients in China but also contribute to the advancement of global cancer treatment strategies.

Following our research, the utilization of sugemalimab as a therapeutic intervention for advanced ESCC yielded results in terms of an ICER of US $61,066.96 per QALY gained. It is noteworthy that this ICER exceeds the WTP threshold of US$39,855.79 per QALY, thereby indicating that sugemalimab does not meet the criteria for being considered a cost-effective treatment option for this specific condition in China.

Sensitivity analysis holds a critical position in both scientific inquiry and decision-making processes, playing a crucial role in examining the impact of changes in input variables on the outcomes of studies and models. Through thorough investigation of the sensitivity of various parameters, researchers can develop a deeper understanding of the interdependent relationships and responsiveness of their findings to individual factors. Notably, a consistent observation emerged that the price of sugemalimab exhibited the most profound influence on the ICER. However, even when modifying the parameters within a range of ±25%, the resulting ICER consistently exceeded the threshold deemed acceptable for WTP. This compelling discovery presents robust evidence to substantiate the claim that the current pricing methodology for sugemalimab is comparably costly and lacks cost-effectiveness in its application to the treatment of advanced ESCC. The PSA result showed that when considering the WTP threshold of US$39855.79 per QALY, there is a mere 1.2% probability of deeming the sugemalimab regimen as a more cost-effective option compared to the placebo group. However, if we take into account a threshold increase to US$63,769.25 per QALY, the sugemalimab regimen is found to be 58.10% more cost-effective than the placebo group.

Currently, there is some research aimed at conducting economic evaluations of immunotherapies for patients with advanced ESCC. The economic evaluation of these treatments is of great importance, as it helps to assess their cost-effectiveness and inform decision-making regarding their inclusion in healthcare systems. For example, a study by Xu et al. used a cost-effectiveness analysis to compare the expenses and benefits of toripalimab *versus* standard chemotherapy for ESCC. The findings of this study indicated that toripalimab plus chemotherapy was likely to be the cost-effective first-line option for patients with advanced ESCC compared with chemotherapy alone (Kang et al., 2024). Another study by Liu et al. focused on evaluating the cost-effectiveness of various second-line immunotherapies for the treatment of ESCC patients. The immunotherapies considered were camrelizumab, nivolumab, pembrolizumab, sintilimab, and tislelizumab. Their results demonstrated that sintilimab might be the optimal treatment alternative for second-line therapy of advanced ESCC in China, followed by tislelizumab and camrelizumab (Liu et al., 2024).

Our study distinguishes itself from previous publications in several significant ways. Firstly, our research utilizes data from a unique clinical trial, the GEMSTONE-304 trial, which sets it apart from previous studies that may have relied on different trial data sources. This inclusion of specific data enhances the credibility and reliability of our findings. Moreover, our analysis incorporates a comprehensive range of factors, such as treatment effects, adverse events, and costs. This multidimensional approach underscores the robustness and thoroughness of our investigation. Additionally, it is noteworthy that no previous publication conducting a cost-effectiveness analysis has specifically utilized data from the GEMSTONE-304 clinical trial. This is an important aspect to highlight as it emphasizes the novelty and originality of our study, as well as its potential to contribute to existing literature in a unique and meaningful way.

We strongly advise against utilizing cost-effectiveness analyses, especially the result of our study, as the sole basis for imposing restrictions on the use of sugemalimab. Instead, we propose employing these analyses to inform policy decisions and enhance access to sugemalimab through improvements in the health insurance system (Fang et al., 2021). We contend that cost-effectiveness analyses can serve as a reliable methodological approach to objectively guide recommendations aimed at reducing the prices of costly drugs. The prevailing exorbitant costs associated with anticancer medications pose an additional financial burden on individuals and the healthcare system as a whole. Recognizing this issue, China has implemented measures such as the centralized national procurement of medicines to alleviate financial strains. Notably, successful negotiations between the government and manufacturers in 2016 resulted in a significant price reduction of gefitinib by over 50%.Drug pricing and national reimbursement negotiation led to a marked decrease in prices and a sharp increase in the utilization of negotiated anticancer medicines (Mingge et al., 2023). The Chinese government is currently engaged in proactive efforts to enhance the availability of novel oncology medications. The accelerated pace of reimbursement decision-making for domestically-produced drugs is perhaps attributed to their competitive pricing advantages as well as the regulator’s endeavors to foster innovation within the domestic pharmaceutical sector (Liu et al., 2022).

Our research aimed to assess the affordability and value of sugemalimab by conducting a comprehensive cost-effectiveness analysis using the ICER. We discovered that reducing the price of sugemalimab by 50% to only $919.92 per 600 mg resulted in an ICER of US$39,547.54 per QALY gained. It is noteworthy that this ICER value closely aligns with the WTP threshold of US$39,855.79 per QALY. These findings underscore the considerable cost-effectiveness of sugemalimab at the lower price. In essence, our results indicate that aligning the ICER of sugemalimab with established WTP thresholds presents an opportunity to expand access to cost-effective treatment for a larger patient population. By strategically reducing the price of sugemalimab, we can enhance the accessibility of this innovative therapy to a broader range of individuals, ultimately maximizing its cost-effectiveness.

This study has the following limitations. Firstly, by relying solely on the GEMSTONE-304 study, there is an increased level of uncertainty in the estimated parameters. The degree of rigor in the conducted trial will inherently impact the reliability of the results obtained. Moreover, as new survival data becomes available, our findings may be subject to potential influences. In light of this, it is imperative to consistently monitor and update these findings.Secondly, it is imperative to acknowledge that in real-world scenarios, the effectiveness of different interventions might be enhanced when used in combination with other treatments, a factor that our model fails to incorporate. Furthermore, our investigation rests on certain assumptions regarding the cost of subsequent treatment options after disease progression. However, in practice, the choice of subsequent treatment regimen will vary based on the specific circumstances of each patient. Encouragingly, our one-way sensitivity analyses demonstrated that even when modifying the estimated range of subsequent treatment regimens, the ICER values consistently exceeded the WTP thresholds, thereby further supporting our conclusions. Lastly, it is important to note that our analysis did not encompass grade 1 or 2 adverse events. We presumed that these events would have a minimal impact on clinical outcomes, and subsequent sensitivity analyses corroborated that adverse drug events do not significantly influence our findings.

## 10 Conclusion

Currently, the utilization of sugemalimab in combination with chemotherapy as a therapeutic approach for the treatment of ESCC in China is not considered cost-effective. However, it is suggested that if the price of sugemalimab is further reduced by 50% to only $919.92 per 600 mg, it may achieving cost-effectiveness in China from the current WTP threshold.

## Data Availability

The original contributions presented in the study are included in the article/Supplementary Material, further inquiries can be directed to the corresponding author.
