# Supplementary material for: Sugemalimab combined with chemotherapy for the treatment of advanced esophageal squamous cell carcinoma: a cost-effectiveness analysis
Source: Front Pharmacol. 2024 Jun 28;15:1396761. doi: 10.3389/fphar.2024.1396761 (PMC11239512; doi:10.3389/fphar.2024.1396761)
Supplement: Supplementary file 1 [file DataSheet1.docx]

**Supplementary Table 1. Comparison of survival models distribution for all population**

|  | AIC | | BIC | |
| --- | --- | --- | --- | --- |
|  | Sugemalimab group | Placebo group | Sugemalimab group | Placebo group |
| PFS |  |  |  |  |
| Weibull | 1657.341 | 795.660 | 1655.102 | 802.068 |
| **Log-logistic** | **1625.689** | **772.385** | **1633.450** | **778.793** |
| Log-normal | 1633.395 | 777.065 | 1464.156 | 783.473 |
| Gompertz | 1691.367 | 824.628 | 1699.128 | 831.036 |
| Exponential | 1701.586 | 831.962 | 1705.466 | 835.166 |
| Gamma | 1644.366 | 793.971 | 1652.127 | 790.380 |
| OS |  |  |  |  |
| Weibull | 1433.714 | 778.419 | 1441.475 | 784.827 |
| **Log-logistic** | **1432.703** | **773.718** | **1440.464** | **780.126** |
| Log-normal | 1438.660 | 775.602 | 1446.421 | 782.010 |
| Gompertz | 1443.425 | 790.034 | 1451.186 | 796.442 |
| Exponential | 1456.657 | 799.094 | 1460.537 | 802.298 |
| Gamma | 1433.379 | 775.809 | 1441.140 | 782.217 |

AIC: Akaike information criterion; BIC: Bayesian Information Criterion; OS: Overall survival; PFS: Progression-free survival;


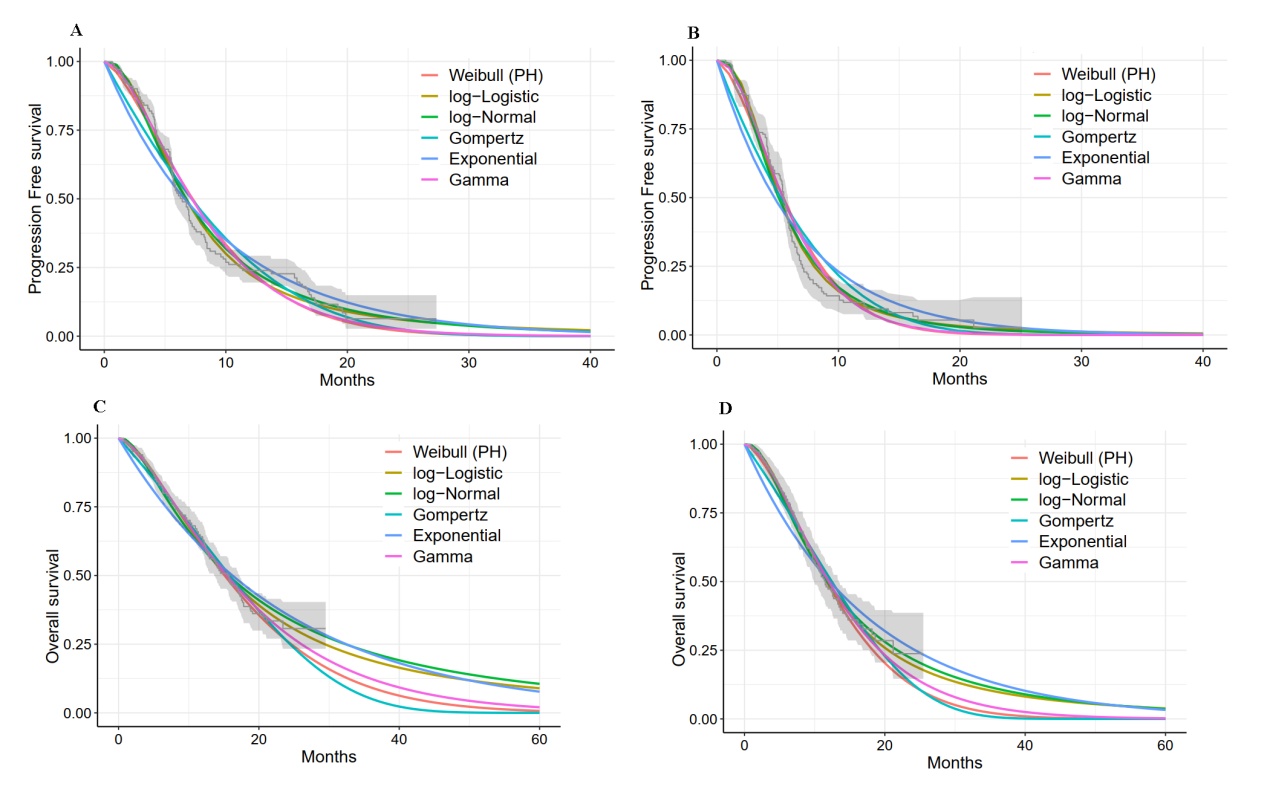


**Supplementary Figure 1.** A: Modes simulation visual progression-free survival curve of sugemalimab group ; B:Modes simulation visual progression-free survival curve of placebo group; C:Modes simulation visual overall survival curve of sugemalimab; D: Modes simulation visual overall survival curve of placebo group
